# Toxicity evaluation of cordycepin and its delivery system for sustained *in vitro* anti-lung cancer activity

**DOI:** 10.1186/s11671-015-0851-1

**Published:** 2015-03-27

**Authors:** Pornanong Aramwit, Supatra Porasuphatana, Teerapol Srichana, Titpawan Nakpheng

**Affiliations:** Bioactive Resources for Innovative Clinical Applications Research Unit and Department of Pharmacy Practice, Faculty of Pharmaceutical Sciences, Chulalongkorn University, PhayaThai Road, Phatumwan, Bangkok 10330 Thailand; Division of Pharmacognosy and Toxicology, Faculty of Pharmaceutical Sciences, KhonKaen University, Mittraphap Road, TambonMuang, KhonKaen 40002 Thailand; Department of Pharmaceutical Technology and Drug Delivery System Excellence Center, Faculty of Pharmaceutical Sciences, Prince of Songkla University, 15 Karnjanavanich Rd, Hat Yai, Songkla 90110 Thailand

**Keywords:** Cordycepin, Gelatin, Nanoparticles, Lung cancer, Toxicity, Sustained release

## Abstract

In the previous study, we have found that the cordycepin which was extracted from *Cordyceps* mycelia produced by growing *Cordyceps militaris* on the dead larva of *Bombyx mori* silkworms showed the anti-proliferative effect toward lung cancer cells without toxicity to non-cancer cells. In this work, the cordycepin was tested for its *in vitro* mutagenicity and *in vivo* toxicity. From the Ames test and subacute toxicity test using oral administration in a rat model, the cordycepin was proved to be a non-mutagenic and non-toxic compound. The hematology and blood chemistry as well as the microanatomical characteristic of the tissues of rats fed with cordycepin every day for consecutive 30 days were comparable to those of the normal ones. Then, the cordycepin was incorporated in gelatin type A (GA) and gelatin type B (GB) nanoparticles aimed to sustain its release and activity. The cordycepin incorporated in both GA and GB nanoparticles showed the sustained release profiles. GA nanoparticles could encapsulate cordycepin at higher encapsulation efficiency due to the attractive electrostatic interaction between the positive-charged GA and the negative-charged cordycepin. However, GA nanoparticles released cordycepin at the higher amount possibly because of the large surface area of small size nanoparticles. Comparing to GB nanoparticles, the higher amount of cordycepin released from GA nanoparticles showed the higher anti-proliferative and anti-migratory effects on A549 lung cancer cells. In conclusion, GA nanoparticles were suggested as a suitable carrier for the sustained release of cordycepin. The GA nanoparticles releasing cordycepin could be an effective and non-invasive material for the treatment of lung cancer cells.

## Background

Lung cancer has recently been the major cause of death worldwide [1]. In 2012, there were 1.59 million deaths due to the lung cancer, accounting for about 20% of cancer-related deaths [2]. Common treatments for lung cancer include surgery, chemotherapy, and radiation therapy [3]. By using chemotherapy, high dose of drugs is required to kill lung cancer cells. Unfortunately, these drugs also exhibit the toxic effect to normal cells and thereby bring the patient to various adverse side effects such as diarrhea, cutaneous rash, or liver and kidney problems [4,5].

Natural compounds that possess anti-cancer activity have received increasing interest to be used as alternative drug. Some botanically derived compounds have potential efficacy in the treatment of lung cancer with minimal side effects [6,7]. Their mechanisms against lung cancer cells including alkylating agents, topoisomerase poisons, DNA synthesis inhibitors, protein synthesis inhibitors, immunoceuticals, and lipoxygenase inhibitors are reported [6]. *Cordyceps* mushroom is a genus of ascomycete fungi that are mainly found on insects and other arthropods [8]. It has been used as traditional Chinese medicine to treat various types of cancer for centuries [9]. The *Cordyceps* consists of cordycepin, nucleosides, and various polysaccharides, however, most of the pharmacologic effects of *Cordyceps* are derived from the cordycepin component. Cordycepin (3-deoxyadenosine, C_10_H_13_N_5_O_3_) is a nucleoside analogue compound that shows selective anti-cancer effects [10-13].

Many studies have focused on the effects of cordycepin on leukemia, melanoma, epithelioid cervix carcinoma, and breast cancer [14,15] while there are only a few studies investigating its action on lung cancer. In our previous work, the cordycepin has been successfully extracted from *Cordyceps* grown on the dead larva of *Bombyx mori* silkworms, and it showed a potent anti-proliferation activity toward human lung adenocarcinoma epithelial A549 cell line [16]. Nevertheless, in order to achieve the sustained activity of cordycepin for the improved therapeutic effects, the delivery system of cordycepin is required. It is known that the treatment of cancer should be minimal invasive to avoid the invasion and spreading of cancer cells to the adjacent site. In this study, the injectable gelatin nanoparticles are introduced as carrier for cordycepin delivery. Previously, we have developed nanoparticles from type A and type B gelatin and suggested them for the delivery of different compounds including methylene blue, eosin, and sericin [17]. It was found that the encapsulation efficiency and release pattern of these compounds from the gelatin nanoparticles depended mainly on their charge characteristic. When selected, the appropriate type of gelatin nanoparticles, the controlled release of compound incorporated could be achieved.

In this study, the cordycepin extracted from *Cordyceps* grown on the dead larva of *B. mori* silkworms was incorporated in type A and type B gelatin nanoparticles. Encapsulation efficiency and *in vitro* release of cordycepin from gelatin nanoparticles were evaluated. The cytotoxic and migration tests of cordycepin released from gelatin nanoparticles against human small airway epithelial cells ((SAEC) a representative of non-cancer cells) and human lung adenocarcinoma epithelial cell line (A549, a representative of lung cancer cells) were performed. Furthermore, the Ames test and *in vivo* subacute toxicity test were carried out to evaluate the mutagenicity and toxicity of the cordycepin, respectively.

## Methods

### Materials

The larvae of *B. mori* strain Chul 3/2 (green-shell cocoons) were kindly supplied by Chul Thai Silk Co., Ltd. (Petchaboon province, Thailand). The spores of *Cordyceps militaris* (NBRC 100741) were purchased from National Institute of Technology and Evaluation (Tokyo, Japan). Type A (GA, pI 9) and type B (GB, pI 5) gelatin were supplied from Nitta Gelatin Inc. (Osaka, Japan). Other chemicals were analytical grade and used without further purification.

### Culture of *Cordyceps* mycelia and extraction of cordycepin

The production of *Cordyceps* and the extraction of cordycepin were carried out following the protocol of our previous work [16]. Briefly, the spores of *C. militaris* were cultured in an artificial diet composed of silk larva, soy sauce, and trehalose dissolved in deionized water for the production of *Cordyceps*. For the extraction of cordycepin, the *Cordyceps* was dissolved in deionized water at 95°C with continuous shaking for 1 h. Then, the sample solutions were filtrated through a 0.22-μm membrane. The cordycepin was obtained.

### Ames test of cordycepin

Mutagenicity of the cordycepin was tested by bacterial reverse mutation assay (Ames test) using *Salmonella typhimurium* strains TA98 (to detect frame shift mutation). Cordycepin was dissolved in sterile water at a concentration of 1 mg/mL and further diluted to 1:1, 1:5, 1:10, and 1:20 dilutions with sterile water. Various concentrations of cordycepin were incubated with TA98 at 37°C for 1 h prior to the addition into melted top agar which contained histidine/biotine (124/96 μg/mL). Positive (50 μg/plate of sodium azide) and negative (phosphate buffer) controls were used to compare the number of revertant colonies. Plates were incubated at 37°C for 48 h. The result was determined based on the number of revertant colonies of TA98 incubated with cordycepin or positive control, comparing with that of the negative control. The experiment was performed in triplicates.

### *In vivo* subacute toxicity test of cordycepin

The animals used in this study was approved by the Ethics Committee of the Faculty of Medicine, Chulalongkorn University (No. 18/57). The animal experiments were performed according to the Chulalongkorn University Animal Care and Use Committee (CU-ACUC). Thirty of the eight-week-old male Wistar rats (weight 200 ± 10 g) were used for the experiment. The rats were fed with a standard diet and housed individually under controlled temperature (23°C to 22°C). For the subacute toxicity test, the rats were divided into three groups as follows, 0.25 mg/mL, 1 mg/mL, and phosphate-buffered saline as a control group (*n* = 10).

Each rat received 1 mL of the above solution in every day for consecutive 30 days. After that, all rats were sacrificed. The blood was collected from the heart. The hematology values (e.g., hemoglobin, hematocrit, erythrocyte count, leucocyte count, and platelet count) and clinical chemistry (e.g., total bilirubin, alkaline phosphatase, total protein, albumin, blood urea nitrogen, creatinine, alanine transaminase, aspartate aminotransferase, uric acid, glucose, triglyceride, and globulin) were assessed. The organs including the liver, kidney, spleen, testis, and brain were collected from each rat, fixed with 10 wt.% formaldehyde solution at room temperature, embedded in paraffin block, sectioned, followed by staining with hematoxylin and eosin (H&E). For liver tissue, the macrovesicular steatosis, sinusoidal congestion, pyknotic nuclei, glycogen storage, and lymphocyte aggregation were evaluated. Glomerulus and collecting duct in the kidney and histology of spleen, testis, and brain tissue were also observed, comparing to the tissue collected from control rats.

### Fabrication of gelatin nanoparticles

The gelatin nanoparticles were fabricated by water in oil (w/o) emulsion technique. Briefly, GA and GB solutions (2.5 wt.%) were slowly dropped into 600 mL of pre-warmed soybean oil, followed by homogenization at 50°C, 3,400 rpm for 15 min. The temperature was decreased to 4°C for the gelation of gelatin nanoparticles. The nanoparticles obtained were washed repeatedly with cold acetone to completely remove residual oil. The air-dried non-crosslinked nanoparticles (1 g) were crosslinked with 0.05 vol.% glutaraldehyde in acetone-to-water (3:1) solution at 4°C for 20 h, followed by washing the residual aldehyde groups with 100 mM aqueous glycine solution for 2 h and washed repeatedly with deionized water. After freeze-drying, the glutaraldehyde-crosslinked GA and GB nanoparticles were obtained.

### Measurement of zeta potential and size of gelatin nanoparticles

GA and GB nanoparticles were homogenously suspended in deionized water (pH 5.5). Zeta potentials and sizes of the GA and GB suspensions were measured by elastic light scattering (ELS, ELS-7000AS instrument, Otsuka Electronics, Tokyo, Japan) at 25°C and electric field strength of 100 V/cm. The zeta potential was automatically calculated using the Smoluchowski equation. The zeta potential of *Cordyceps* in solution was also measured (*n* = 3).

### Entrapment and loading efficiencies of cordycepin in gelatin nanoparticles

GA and GB nanoparticles (50 mg) were incubated with 1 mL cordycepin solution (1.2 mg/mL) at 4°C for 48 h for physical absorption. After air drying, the cordycepin-encapsulated nanoparticles were obtained. To evaluate the encapsulation and loading efficiencies of cordycepin in the nanoparticles, the nanoparticles were hydrolyzed with 6 N hydrochloric acid. The concentration of cordycepin (total concentration of protein subtracted with the concentration of gelatin nanoparticles) in the supernatant was measured by the bicinchoninic acid (BCA) protein assay kit [18]. The amount of cordycepin was determined from the standard curves prepared from various concentrations of cordycepin. The entrapment efficiency and drug loading percentage of cordycepin were calculated according to following equations:

$$ \mathrm{Entrapment}\ \mathrm{efficiencies}\ \left(\mathrm{E}\mathrm{E},\ \%\right) = \left({C}_{\mathrm{E}}/{C}_0\right) \times 100 $$

$$ \mathrm{Drug}\ \mathrm{loading}\ \mathrm{percentage}\ \left(\mathrm{L}\mathrm{E},\ \%\right) = \left({C}_{\mathrm{E}}/{C}_{\mathrm{MS}}\right) \times 100 $$

where *C*_0_ and *C*_E_ represent the amounts of cordycepin loaded and entrapped in the nanoparticles, respectively, while *C*_MS_ represents the amount of nanoparticles (*n* = 3).

### *In vitro* release test

The cordycepin-encapsulated nanoparticles were placed in 10 mL phosphate-buffered saline (PBS) at 37°C under shaking. At the pre-determined time points, the supernatant was collected and replaced with same volume of fresh PBS solution. The concentration of cordycepin released into the supernatant was measured by BCA assay as described previously [18]. The percentages of cumulative release of cordycepin from the nanoparticles were calculated (*n* = 3).

### *In vitro* cytotoxic test of cordycepin released from nanoparticles

SAEC (CC-2547, Cambrex, BioScience, Walkersville, MD, USA) were used as a representative of non-cancer cells. The cells were seeded on the 96-well plates at a density of 1 × 10^5^ cells/well and cultured in small airway epithelial cells basal medium (SABM) supplemented with growth factors supplied in the SAGM SingleQuot kit (Lonza™ Inc., Walkersville, MD, USA) at 37°C, 5% CO_2_. At 1 day after seeding, the medium was removed and refreshed with fresh medium containing the GA or GB nanoparticles encapsulating cordycepin (0, 10, 20, 30, and 40 μM). The cordycepin solution (5 μM) was used as a positive control. The viability of cells cultured for 24 h was quantified using the conventional 3-(4,5-dimethylthiazol-2-yl)-2,5-diphenyltetrazolium bromide (MTT) assay (*n* = 4) [19].

Human lung adenocarcinoma epithelial cell line, A549 cells (ATCC CCL-185, Rockville, MD, USA), was used as a representative of lung cancer cells. The cells were cultured in Ham’s F-12K (Kaighn’s) medium supplemented with 10% FBS and 100 U/mL penicillin-streptomycin. The cell seeding and culturing with the GA or GB nanoparticles encapsulating cordycepin (0, 10, 20, 30, and 40 μM cordycepin) were performed as the earlier described. The viability of cells cultured for 24 h was quantified by MTT assay.

### Migration assay

*In vitro* migration assay was performed to evaluate the anti-migratory activity of cordycepin released from gelatin nanoparticles toward human lung A549 cell line. The migration assay was carried out according to a modified Boyden’s chamber assay using six-well transwell culture plates with 8-μm-pore size-polycarbonate filter (Corning/Fisher Scientific, Schwerte, Germany) [20]. Briefly, A549 cells were seeded in the upper chamber (1 × 10^6^ cells/well in 1.5 mL). The same media containing GA or GB nanoparticles encapsulating cordycepin (0, 10, 20, 30, and 40 μM cordycepin) 2.6 mL was added into the lower chamber. Cells were incubated at 37°C, 5% CO_2_ for 24 h. The cordycepin solution (5 μM) was used as a positive control. The number of migrated cells was counted by using light microscope at high magnification field (20×).

### Statistical analysis

All quantitative data were shown as mean ± standard deviation. The statistical significance was determined by paired and unpaired Student’s *t*-tests along with ANOVA. A value of *p* < 0.05 was considered to be significant.

## Results and discussion

In our recent work, we have found that the *Cordyceps* mycelia could be produced by growing *C. militaris* on the dead larva of *B. mori* silkworms [16]. The cordycepin with anti-cancer activity but non-toxicity to non-cancer cells was extracted from this *Cordyceps*. We have previously shown that the cordycepin extracted had an anti-proliferative potential toward human non-small cell lung cancer NCI-H460 cells and human lung adenocarcinoma epithelial A549 cell line due to the disruption of cell membrane by cordycepin [16]. In this study, we aimed to prolong the action of cordycepin on lung cancer cells, the delivery system of cordycepin was therefore introduced. Before the cordycepin was combined with the delivery system, it was tested for the *in vitro* mutagenicity and *in vivo* toxicity. The number of revertant colonies of TA98 after tested with cordycepin at various dilutions (1:1, 1:5, 1:10, and 1:20) varied between 41 and 61, accounting for 1.19 to 1.81 times of the values observed in the phosphate buffer group (negative control), as shown in Table 1. No concentration-related increase of number of revertant colonies treated with cordycepin was observed. This indicated the lack of direct mutagenic effect of cordycepin on TA98 bacteria and would imply that cordycepin is a non-mutagenic agent. On the other hand, the positive control (sodium azide) showed significant increase in the revertant colony number (656 colonies) which was calculated as 19.29 times of the values of negative control. This confirmed the validity of the Ames method which is considered as the first test for all regulatory genetic toxicity testing [21].

**Table 1:** **The number of revertant colonies of TA98 after being tested with cordycepin at various dilutions**

**Group**	**Dilution**			
	**No dilution**	**1:5**	**1:10**	**1:20**
Phosphate buffer	34	**-**	**-**	**-**
Sodium azide	656 (19.29)	**-**	**-**	**-**
Cordycepin	42 ± 7 (1.23)	41 ± 15 (1.19)	61 ± 23 (1.81)	54 ± 4 (1.57)

Mean ± SD, *n* = 3.

**Figure 1:**
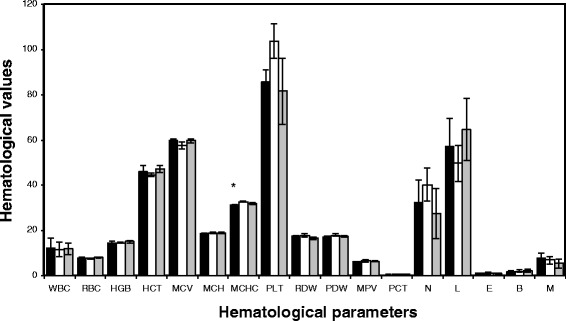
**Hematology values in the blood of rats fed with cordycepin every day for consecutive 30 days.** Groups: (filled square) 0.25 mg/mL, (empty square) 1 mg/mL, (gray square) control. *ANOVA *p* value, *p* < 0.05. WBC, white blood cell; RBC, red blood cell; HGB, hemoglobin; HCT, hematocrit; MCV, mean corpuscular volume; MCH, mean corpuscular hemoglobin; MCHC, mean cell hemoglobin; PLT, platelet; RDW, red cell distribution width; PDW, platelet distribution width; MPV, mean platelet volume; PCT, plateletcrit; N, neutrophil; L, lymphocyte; E, eosinophil; B, basophil; M, monophil.

**Figure 2:**
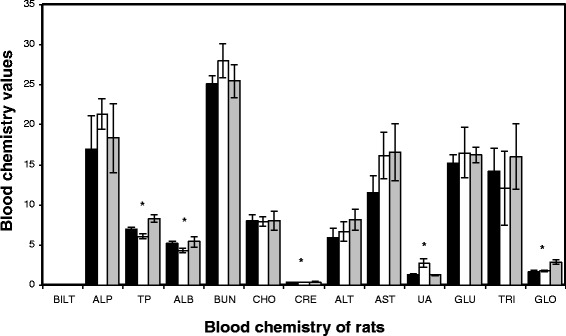
**Clinical chemistry in the blood of rats fed with cordycepin every day for consecutive 30 days.** Groups: (filled square) 0.25 mg/mL, (empty square) 1 mg/mL, (gray square) control. *ANOVA *p* value, *p* < 0.05. BILT, total bilirubin; ALP, alkaline phosphatase; TP, total protein; ALB, albumin; BUN, blood urea nitrogen; CRE, creatinine; ALT, alanine transaminase; AST, aspartate aminotransferase; UA, uric acid; GLU, glucose; TRI, triglyceride; GLO, globulin.

The *in vivo* subacute toxicity of cordycepin was evaluated in a rat model using oral administration. The hematology values in the blood of rats fed with cordycepin (0.25 and 1 mg/mL) are presented in Figure 1. The values of white blood cell, red blood cell, hemoglobin, hematocrit, mean corpuscular volume, mean corpuscular hemoglobin, platelet, red cell distribution width, platelet distribution width, mean platelet volume, neutrophil, lymphocyte, eosinophil, basophil, and monophil were comparable to the values of control rats fed with phosphate buffer. Although the mean cell hemoglobin values of rats fed with cordycepin were significantly different from those of the control rats, they were in the range of normal blood (30 to 38 g/dL). The values of clinical chemistry in the blood of rats fed with cordycepin are shown in Figure 2. The values of total bilirubin, alkaline phosphatase, blood urea nitrogen, alanine transaminase, aspartate aminotransferase, glucose, and triglyceride seemed to be normal when compared to the values of control rats. It is noticed that there was a significant difference in total protein (which is the sum of albumin and globulin) between the rats fed with cordycepin and the control rats. However, all values were in the normal range (6.0 to 8.3 g/dL). We also found that the values of creatinine and uric acid, which indicated the kidney function, of the rats fed with cordycepin were somehow different from those of control rats. However, the blood urea nitrogen values showed the normal function of the kidney in all rats.

**Figure 3:**
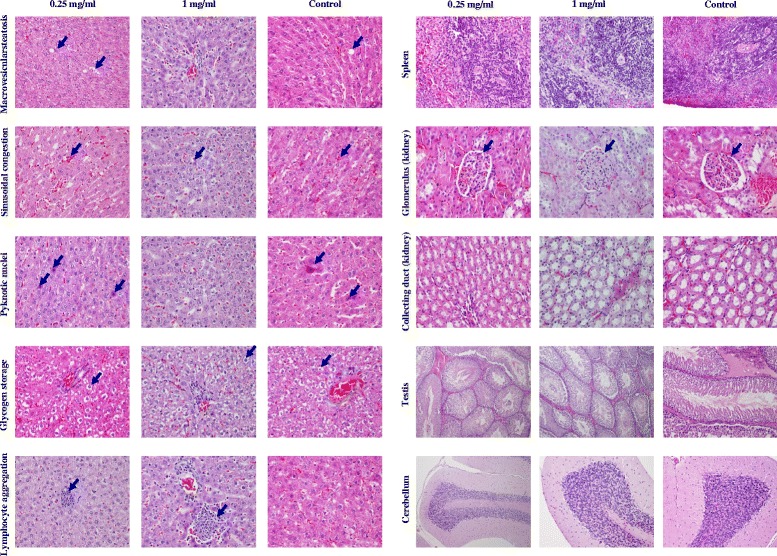
**Histology of tissue collected from different organs.** The liver, spleen, kidney, testicle, and brain of the rats fed with cordycepin every day for consecutive 30 days.

Furthermore, the tissue was collected from different organs (the liver, spleen, kidney, testis, and brain) of rats and their microanatomical characteristics were analyzed, as shown in Table 2 and Figure 3. The histology of tissue collected from the liver, spleen, kidney, testis, and brain was similar to those of the normal tissue, although some pyknotic nuclei and lymphocyte aggregation in the liver of rats treated with cordycepin were found to be different. However, it is supposed that this effect would arise from the normal physiological change of rats rather than the cordycepin treatment effect. In overall, these results indicated that the cordycepin extracted did not show any signs of sub-acute toxicity as reported elsewhere [22].

**Table 2:** **Microanatomical characteristic of tissue collected from different organs**

**Organ**	**Microanatomical characteristic of tissue**	**Percentage of characteristic founded (%)**			***p*** **value; Chi-square**
		**Group**			
		**0.25 mg/ml**	**1 mg/ml**	**Control**	
Liver	Macrovesicularsteatosis	33.3 (3/9)	0 (0/7)	37.5 (3/8)	0.189
	Sinusoidal congestion	100 (9/9)	100 (7/7)	100 (8/8)	-
	Pyknotic nuclei	88.9 (8/9)	0 (0/7)*	62.5 (5/8)	0.002
	Glycogen storage	77.8 (7/9)	71.4 (5/7)	75 (6/8)	0.959
	Lymphocyte aggregation	11.1 (1/9)	57.1 (4/7)*	0 (0/8)	0.016
Spleen	Normal	100 (9/9)	100 (7/7)	100 (8/8)	-
Kidney	Normal	100 (9/9)	100 (7/7)	100 (8/8)	-
Testis	Normal	100 (9/9)	100 (7/7)	100 (8/8)	-
Brain	Normal	100 (9/9)	100 (7/7)	100 (8/8)	-

The liver, spleen, kidney, testis, and brain of rats fed with cordycepin every day for consecutive 30 days; *indicates significant difference compared to control (*p* < 0.05).

After that, the cordycepin was incorporated into the delivery system. In this study, the gelatin nanoparticles in which we have previously developed were introduced [17]. The gelatin nanoparticles were selected as the release carrier because they had large surface area per unit mass for drug encapsulation [23]. Moreover, the nanoparticles could be applied as injectable material which is considered as non-invasive route for the treatment of patient [24]. Due to the difference in charge of type A (GA) and type B gelatin (GB), GA and GB nanoparticles might show different effects on the encapsulation efficiency and release behavior of compound incorporated. To compare this, both GA and GB nanoparticles were studied as the release carrier of cordycepin. Table 3 presents the characteristics of the GA and GB nanoparticles and cordycepin. The average size of GB nanoparticles (1,073.78 nm) was significantly larger than that of GA nanoparticles (540.05 nm). Both GB nanoparticles and cordycepin showed negative charges (−12.18 and −24.9 mV, respectively) while the GA nanoparticles showed positive charge (+11.75 mV).

**Table 3:** **Characteristics of the GA and GB nanoparticles and cordycepin**

**Sample**	**Size (nm)**	**Zeta potential (mV)**	**Entrapment efficiency (%)**	**Loading percentage (%)**
GA	540.05 ± 83.74	+11.75 ± 0.38	61.11 ± 9.62	1.46 ± 0.23
GB	1,073.78 ± 238.63*	−12.18 ± 1.20	25.00 ± 8.33*	0.60 ± 0.20
Cordycepin	N/A	−24.9 ± 0.30	N/A	N/A

*p < 0.05, significant against the values of GA nanoparticles. Mean ± SD, *n* = 3. N/A, not applicable.

**Figure 4:**
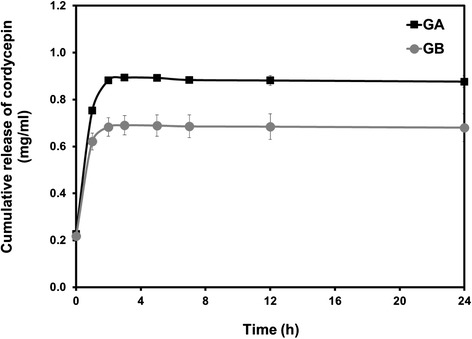
***In vitro*** **cumulative release of cordycepin from GA and GB nanoparticles.** After incubated in phosphate buffered saline (pH 7.4) at 37°C for different periods.

When loaded cordycepin into nanoparticles, the encapsulation efficiency of cordycepin in GA nanoparticles (61.11%) was significantly higher than that of GB nanoparticles (25.00%), as can be seen in Table 3. This might be the result of the attractive electrostatic interaction between the positive-charged GA nanoparticles and the negative-charged cordycepin. The phenomenon of attractive electrostatic interaction between carrier and compound incorporated was also reported by Okhawilai et al. [25]. It is noted that the loading percentages of cordycepin in both GA and GB nanoparticles were very low, and the significant difference was not observed. When subjected into the phosphate buffer medium (pH 7.4), the sustained release profiles of cordycepin released from both GA and GB nanoparticles were obtained (Figure 4). It is interesting that the cordycepin was released from GA nanoparticles at the higher extent along the test period although they had the attractive electrostatic interaction. It is possible that GA nanoparticles had significantly smaller size than the GB ones and may show the dramatically large surface area that accelerated the release of cordycepin. In addition, the accelerated released of cordycepin might come from the faster degradation rate of GA nanoparticles than that of GB nanoparticles, as reported previously [17].

**Figure 5:**
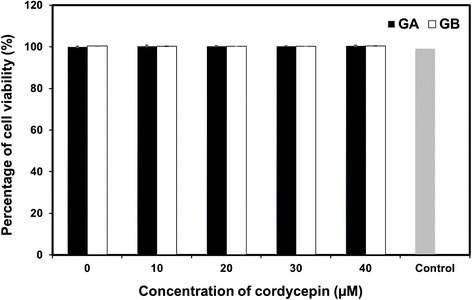
***In vitro*** **cytotoxicity of the GA and GB nanoparticles.** Releasing cordycepin against SAEC after being cultured for 24 h; (mean ± SD, *n* = 4), control = cordycepin solution (5 μM).

**Figure 6:**
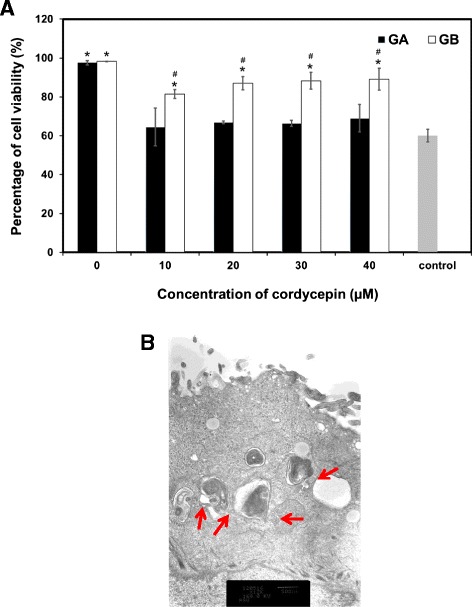
***In vitro*** **cytotoxicity of the GA and GB nanoparticles and morphology of A549 cells. (A)** *In vitro* cytotoxicity of the GA and GB nanoparticles releasing cordycepin against A549 cells after being cultured for 24 h. (mean ± SD, *n* =4), control = cordycepin solution (5 μM). **(B)** Morphology of A549 cells after treating with GA nanoparticles releasing 40 μM cordycepin for 24 h, observed on TEM (arrow: disrupted cell membrane).

The GA and GB nanoparticles releasing cordycepin were cultured with SAEC to test their cytotoxicity to non-cancer cells. It was shown that any concentration of cordycepin incorporated into nanoparticles was not toxic to SAEC. The viability of SAEC remained 95% to 100% even cultured with the nanoparticles releasing cordycepin for 1 day (Figure 5). After that, the toxicity of cordycepin released from GA and GB nanoparticles toward lung cancer cells (A549 cells) was tested. The results showed that the cordycepin released from GA nanoparticles potentially killed A549 cells comparable to the positive control (5 μM cordycepin solution). Percentage of viability of A549 cells treated with GA nanoparticles releasing cordycepin was significantly lower than those of GB nanoparticles (Figure 6A). This might be corresponded to the release profile of cordycepin as shown in Figure 4. The higher amount of cordycepin released from GA nanoparticles would show stronger anti-proliferative effect on A549 cells than that of GB nanoparticles. It is noted that the anti-proliferative effect of cordycepin on A549 cells was not in a dose-dependent manner, possibly due to the sustained concentration of cordycepin released from the nanoparticles. Morphology of A549 cells after treated with GA nanoparticles releasing 40 μM cordycepin observed on TEM was elucidated in Figure 6B. It showed that some cells were damaged at the epithelia resulting in the release of the cytoplasm. Our data also confirmed the *in vitro* cytotoxic effect of cordycepin toward cancer cells, corresponding to that reported elsewhere [26-28].

**Figure 7:**
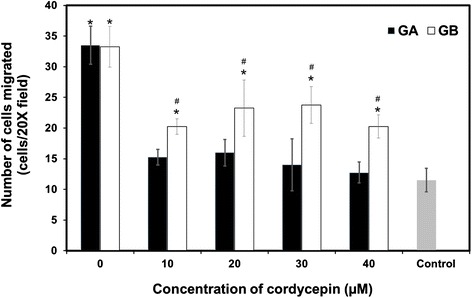
***In vitro*** **migration of A549 cells incubated with GA and GB nanoparticles.** Releasing cordycepin for 24 h; (mean ± SD, *n* = 4), control = cordycepin solution (5 μM).

Finally, the anti-migratory effect of cordycepin against A549 cells was evaluated. Again that, the numbers of A549 cells migrated into the lower chamber filled with GA nanoparticles releasing cordycepin were significantly lower than those of GB nanoparticles releasing cordycepin at any cordycepin concentration (Figure 7). This elucidated the higher anti-migratory effect of the GA nanoparticles releasing cordycepin on A549 cells than the GB nanoparticles releasing cordycepin due to the higher amount of cordycepin released. The anti-migratory effect of cordycepin against cancer cells was also reported previously [29,30]. All the results above confirmed that GA nanoparticles acted as a potential carrier for the sustained release of cordycepin while the high amount of cordycepin released from GA nanoparticles showed the high anti-proliferative and anti-migratory effects on A549 lung cancer cells.

## Conclusions

The cordycepin was extracted from *Cordyceps* mycelia produced by growing *C. militaris* on the dead larva of *B. mori* silkworms according to our previous study. From *in vitro* mutagenicity and *in vivo* subacute toxicity tests, the cordycepin was proved to be a non-mutagenic and non-toxic compound. The cordycepin incorporated in GA and GB nanoparticles showed the sustained release profiles. GA nanoparticles could encapsulate cordycepin at higher encapsulation efficiency due to the attractive electrostatic interaction, however, GA nanoparticles released higher amount of cordycepin because of the large surface area of small size nanoparticles. The high amount of cordycepin released from GA nanoparticles showed potential anti-proliferative and anti-migratory effects on A549 lung cancer cells. Therefore, we suggested GA nanoparticles releasing cordycepin as an effective and non-invasive material for the treatment of lung cancer cells.

## References

[1] Malvezzi M, Bosetti C, Rosso T, Bertuccio P, Chatenoud L, Levi F (2013). Lung cancer mortality in European men: trends and predictions. Lung Cancer.

[2] De Martel C, Ferlay J, Franceschi S, Vignat J, Bray F, Forman D (2012). Global burden of cancers attributable to infections in 2008: a review and synthetic analysis. Lancet Oncol.

[3] Ferrell B, Koczywas M, Grannis F, Harrington A (2011). Palliative care in lung cancer. Surg Clin N Am.

[4] D’Antonio C, Passaro A, Gori B (2014). Bone and brain metastasis in lung cancer: recent advances in therapeutic strategies. Ther Adv Med Oncol.

[5] D’Arcangelo M, Hirsch FR (2014). Clinical and comparative utility of afatinib in non-small cell lung cancer. Biol Targets Ther.

[6] Ancuceanu RV, Istudor V (2004). Pharmacologically active natural compounds for lung cancer. Altern Med Rev.

[7] Gomathinayagam R, Sowmyalakshmi S, Mardhatillah F, Kumar R, Akbarsha MA, Damodaran C (2008). Anticancer mechanism of plumbagin, a natural compound, on non-small cell lung cancer cells. Anticancer Res.

[8] Holliday J, Cleaver M (2008). Medicinal value of the caterpillar fungi species of the genus Cordyceps (Fr.) link (Ascomycetes): a review. Int J Med Mushr.

[9] Mizuno T (1999). Medicinal effects and utilization of Cordyceps (Fr.) link (Ascomycetes) and Isaria Fr. (Mitosporic fungi) Chinese caterpillar fungi, ‘Tochukaso’. Int J Med Mushr.

[10] Rottenberg ME, Masocha W, Ferella M, Pe-titto-Assis F, Goto H, Kristensson K (2005). Treatment of african trypanosomiasis with cordycepin and adenosine deaminase inhibitors in a mouse model. J Infect Dis.

[11] Xu FL, Lee YL, Tsai WY, Lin SJ, Yang ZQ, Yang CC (2005). Effect of cordycepin on hantaan virus 76–118 infection of primary human embryonic pulmonary fibroblasts: characterization of apoptotic effects. Acta Virol.

[12] Cho HJ, Cho JY, Rhee MH, Park HJ (2007). Cordycepin (3′-deoxyadenosine) inhibits human platelet aggregation in a cyclic AMP- and cyclic GMP-dependent manner. Eur J Pharmacol.

[13] Nakamura K, Yoshikawa N, Yamaguchi Y, Kagota S, Shinozuka K, Kunitomo M (2006). Antitumor effect of cordycepin (3′-deoxyadenosine) on mouse melanoma and lung carcinoma cells involves adenosine a3 receptor stimulation. Anticancer Res.

[14] Foss FM (2000). Combination therapy with purine nucleo-side analogs. Oncology (Williston Park).

[15] Thomadaki H, Tsiapalis CM, Scorilas A (2005). Poly- adenylate polymerase modulations in human epithelioid cervix and breast cancer cell lines, treated with etoposide or cordycepin, follow cell cycle rather than apoptosis induction. Biol Chem.

[16] Aramwit P, Bang N, Ratanavaraporn J, Nakpheng T, Srichana T (2014). An anti-cancer cordycepin produced by Cordyceps militaris growing on the dead larva of Bombyx mori silkworm. J Agr Sci.

[17] Aramwit P, Jaichawa N, Ratanavaraporn J, Srichana T (2015). A comparative study of type A and type B gelatin nanoparticles as the controlled release carriers for different model compounds. Mat Express.

[18] Schoel B, Welzel M, Kaufmann SHE (1995). Quantification of protein in dilute and complex samples: modification of the bicinchoninic acid assay. J Biochem Biophys Methods.

[19] Mosmann T (1983). Rapid colorimetric assay for cellular growth and survival: application to proliferation and cytotoxicity assays. J Immunol Methods.

[20] Dai X, Tan Y, Cai S, Xiong X, Wang L, Ye Q (2011). The role of CXCR7 on the adhesion, proliferation and angiogenesis of endothelial progenitor cells. J Cell Mol Med.

[21] Kirkland D, Zeiger E, Madia F, Raffaella C (2014). Can *in vitro* mammalian cell genotoxicity test results be used to complement positive results in the Ames test and help predict carcinogenic or *in vivo* genotoxic activity? II. Construction and analysis of a consolidated database. Mutat Res-Gen Tox En.

[22] Meena H, Singh KP, Negi PS, Ahmed Z (2013). Sub-acute toxicity of cultured mycelia of Himalayan entomogenous funfus *Cordyceps sinensis* (Berk.) SACC in rats. Indian J Exp Biol.

[23] Jong WHD, Borm PJA (2008). Drug delivery and nanoparticles: applications and hazards. Int J Nanomedicine.

[24] Wang W, Deng L, Huang P, Xu S, Li X, Lv N, et al. Toxicity and *in vivo* biological effect of the nanoparticular self-supported hydrogel of a thermosensitive copolymer for noninvasive drug delivery. J Biomed Mater Res Part A. 2013. doi:10.1002/jbm.a.34694. 10.1002/jbm.a.34694 23475810

[25] Okhawilai M, Rangkupan R, Kanokpanont S, Damrongsakkul S (2010). Preparation of Thai silk fibroin/gelatin electrospun fiber mats for controlled release applications. Int J Biol Macromol.

[26] Nakamura K, Shinozuka K, Yoshikawa N (2015). Anticancer and antimetastatic effects of cordycepin, an active component of Cordyceps sinensis. J Pharmacol Sci.

[27] Chou SM, Lai WJ, Hong TW, Lai JY, Tsai SH, Chen YH (2014). Synergistic property of cordycepin in cultivated Cordyceps militaris-mediated apoptosis in human leukemia cells. Phytomedicine.

[28] Lee SY, Debnath T, Kim SK, Lim BO (2013). Anti-cancer effect and apoptosis induction of cordycepin through DR3 pathway in the human colonic cancer cell HT-29. Food Chem Toxicol.

[29] Lee EJ, Kim WJ, Moon SK (2010). Cordycepin suppresses TNF-alpha-induced invasion, migration and matrix metalloproteinase-9 expression in human bladder cancer cells. Phytother Res.

[30] Jeong JW, Jin CY, Park C, Han MH, Kim GY, Moon SK (2012). Inhibition of migration and invasion of LNCaP human prostate carcinoma cells by cordycepin through inactivation of Akt. Int J Oncol.

